# Bilateral multiple choroidal granulomas and systemic vasculitis as presenting features of tuberculosis in an immunocompetent patient

**DOI:** 10.1186/s12348-016-0109-9

**Published:** 2016-10-26

**Authors:** Nitin Kumar, Sridharan Sudharshan, Sudha K. Ganesh, Gopal Lingam, Jyotirmay Biswas

**Affiliations:** Medical and Vision Research Foundations, Sankara Nethralaya, 18, College Road, Chennai, 600006 India

**Keywords:** Anti-tubercular therapy, Extra pulmonary tuberculosis, Multiple choroidal granulomas, MPB64 genome, Systemic vasculitis, Polymerase chain reaction

## Abstract

**Background:**

Multiple choroidal granulomas are a rare presentation of tuberculosis. Choroidal granulomas in immunocompetent patients can pose difficulty in diagnosis as in most cases systemic examination may not reveal any evidence of tuberculosis. We report a case of bilateral multiple choroidal granulomas with systemic vasculitis-like features and disseminated tuberculosis in an immunocompetent patient without pulmonary involvement.

**Findings:**

A 26-year-old male Indian patient presented with bilateral blurred vision and systemic illness with vasculitis-like features. Examination revealed bilateral multiple choroidal granulomas and multisystem involvement without pulmonary involvement. Aqueous tap was positive for mycobacterium by polymerase chain reaction along with tissue biopsy leading to diagnosis. There was a good systemic and ocular response to anti-tubercular therapy with resolution of lesions.

**Conclusions:**

Our case emphasizes that, although uncommon, tuberculosis can involve multiple organs without pulmonary involvement and may mimic systemic vasculitis, it is not mandatory to have pulmonary findings for a confirmation of tuberculosis. Timely diagnosis with appropriate treatment can improve systemic and ocular disease.

## Introduction

Tuberculosis primarily affects the lungs but can also have extrapulmonary involvement such as ocular tuberculosis [1]. The proportion of extrapulmonary tuberculosis is on the rise especially in immunocompromised patients [2]. The primary lesions in TB choroiditis are choroidal tubercles which can be multiple, ill defined, round to oval, greyish white, or yellowish deep lesions and indicate hematogenous dissemination of the bacilli [2]. Extrapulmonary tuberculosis can occur either in association with clinically apparent pulmonary disease or uncommonly in isolation. Although multiple organ involvement due to tuberculosis is uncommon in immunocompetent patients [3], they usually occur in association with pulmonary involvement. We report a rare case of an immunocompetent patient presenting with bilateral choroidal granulomas with systemic vasculitis-like features without pulmonary involvement which resolved completely with anti-tubercular treatment.

## Findings

A 26-year-old male presented to us with history of decreased vision in the right eye since 3 months which was gradual in onset and painless in nature. He had complaints of recent loss of weight, malaise, and back pain. He also gave a history of recurrent episodes of severe cramping pain in his calf region and swellings on his palm and nose.

**Fig. 1:**
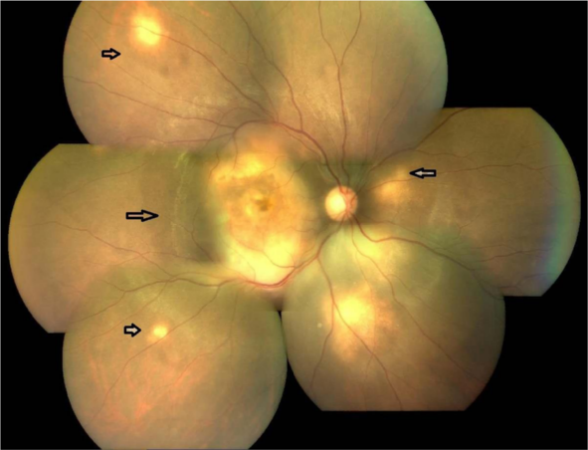
The right eye fundus photograph showing multiple choroidal granulomas affecting macula

**Fig. 2:**
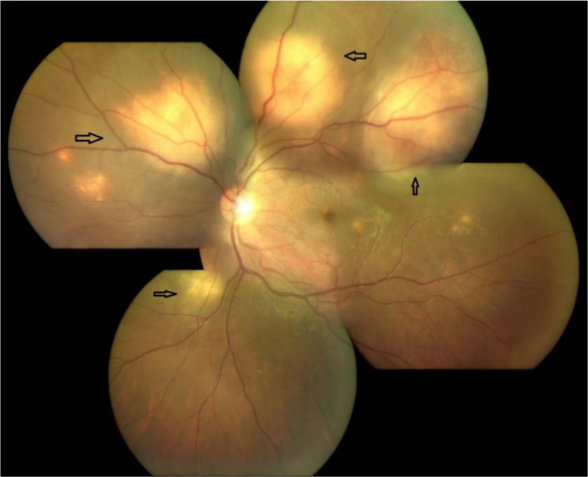
The left eye fundus photograph showing multiple sub-retinal granulomas sparing macula

**Fig. 3:**
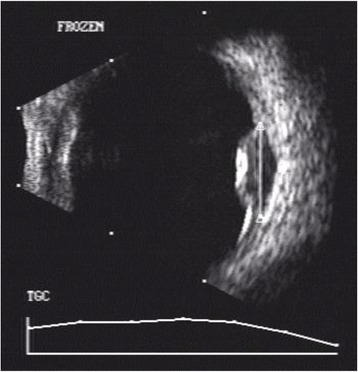
The USG right eye showing presence of sub-retinal abscess

On ophthalmic examination, his best corrected visual acuity (BCVA) was counting fingers close to face in the right eye and 6/6, N6 in the left eye. Slit lamp examination of the right eye revealed an anterior chamber reaction of 2+ cells and vitreous cells while anterior segment was quiet in the left eye. Fundus examination showed presence of multiple yellowish colored sub-retinal lesions in the both eyes suggestive of choroidal granulomas (Figs. 1 and 2). Ultrasonography of the right eye showed a dome-shaped sub-retinal mass in the macular region with retinal detachment adjacent to the mass (Fig. 3). A provisional diagnosis of multiple choroidal granulomas with systemic involvement was considered.

**Fig. 4:**
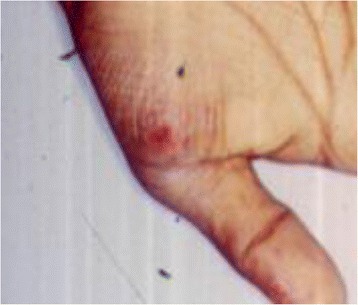
Nodular lesion on the palm

Systemic examination of patient under care of an internist revealed presence of nodular lesions on the palm and nose (Fig. 4). A possible diagnosis of systemic vasculitis or infective endocarditis was also considered. Radiology of the spine revealed evidence of Pott’s spine.

**Fig. 5:**
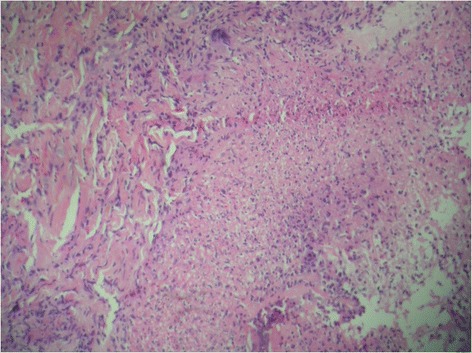
Histopathology of aspirate from nodular eruption on the palm showing the presence of multiple atypical mycobacteria with background presence of inflammatory cell collection suggestive of necrotising inflammation

Blood investigations to rule out systemic vasculitis (antinuclear antibodies, RA factor, c-ANCA, and p-ANCA) and other infective etiologies including HIV were all negative. PPD testing revealed an induration of 10 × 20 mm although high-resolution computed tomography (HRCT) chest was within normal limits. Biopsy and histopathology of the lesions on the palm and nose showed the presence of sub-acute necrotizing inflammation with atypical mycobacterium tuberculosis (Fig. 5).

Anterior chamber tap was positive for MPB 64 genome by polymerase chain reaction. A final diagnosis of multiple choroidal granulomas with systemic vasculitis of tubercular etiology was made. Patient was treated with a four-drug anti-tubercular treatment regimen, consisting of isoniazid, ethambutol, pyrazinamide, and rifampicin for 3 months, followed by isoniazid and rifampicin for 6 months. A tapering dosage schedule of systemic steroids (1 mg/kg body weight) was also advised for a period of 10 weeks.

**Fig. 6:**
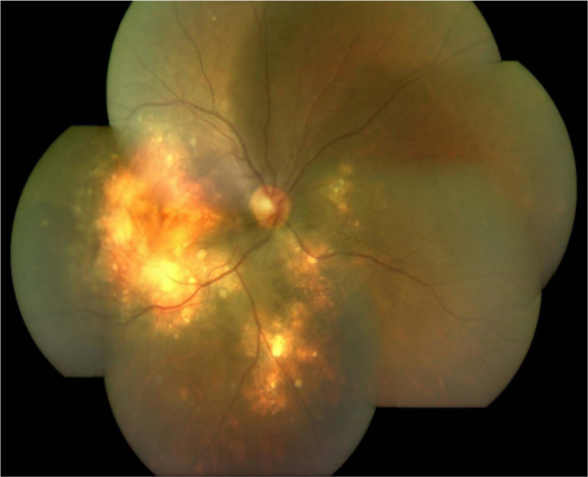
The right eye fundus photograph showing healed granulomas

**Fig. 7:**
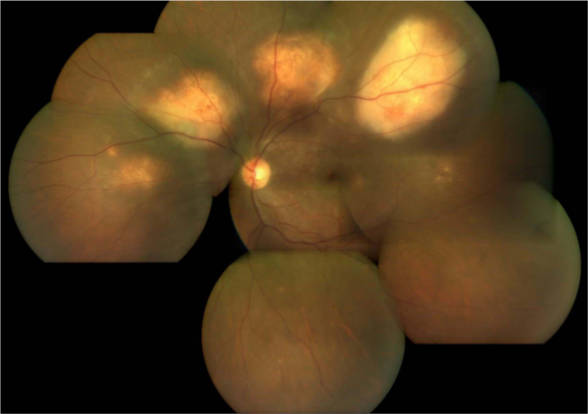
The left eye fundus photograph showing healed granulomas

The ocular lesions and systemic status showed signs of resolution during the follow-up. At 1-year follow-up, fundus lesions resolved completely with resolution of vasculitis. General health of the patient also improved with complete resolution of systemic signs and symptoms without any recurrences (Figs. 6 and 7).

## Discussion

Choroidal involvement is the most common ocular manifestation in patients with pulmonary and systemic tuberculosis [4]. Ocular involvement in disseminated tuberculosis is seen commonly with choroidal tubercles [5]. A rare case of bilateral multiple choroidal granulomas with splenic involvement and miliary tuberculosis has been reported which also had pulmonary involvement [6]. The analysis of the aqueous in their case did not provide any clue to the confirmation of tubercular choroidal granuloma [6]. However, in our case; aqueous tap proved the diagnosis by PCR for MPB 64 which was positive. Histopathologic confirmation for definite diagnosis of choroidal tuberculomas maybe difficult and diagnosis is usually presumptive. In our case, the systemic diagnosis was confirmed by histopathology of the skin lesions.

Choroidal tubercles in an immunocompetent patient can pose difficulty in diagnosis as in most cases systemic examination may not reveal any evidence of tuberculosis and diagnosis is presumptive only. Pulmonary tuberculosis is the commonest systemic manifestation of the disease but without radiological evidence of pulmonary involvement or acid fast bacilli positivity in sputum, confirmation of ocular tuberculosis becomes difficult. It is an uncommon presentation to have disseminated disease without pulmonary involvement [6].

Diagnosis becomes more difficult if ancillary investigations are also negative. In our case, the clinical picture of choroidal granulomas with positive aqueous tap confirmed ocular tuberculosis. The features of systemic vasculitis and biopsy proven nodular lesions on palms which resolved with ATT also indicated disseminated tuberculosis. Significantly, in our case, there was no clinical or radiological evidence of pulmonary tuberculosis. Atypical mycobacterium infections can present with subcutaneous nodules that can be difficult to differentiate from vasculitis, panniculitis, or abscesses [7]. In our case, systemic vasculitis which was initially suspected by the internist to be of inflammatory origin was later proven to be tuberculous by clinical and lab investigations.

This case represents a rare presentation of multiple bilateral tuberculous granulomas as presenting feature of disseminated tuberculosis proven micriobiologically and by histopathology, in an immunocompetent individual especially without pulmonary involvement. Medline search did not reveal any such similar reports to the best of our knowledge.

## Abbreviations

HIV: Human immunodeficiency virus
HRCT: High-resolution computed tomography
PPD: Purified protein derivative
